# Understanding Long-Term Trajectories in Web-Based Happiness Interventions: Secondary Analysis From Two Web-Based Randomized Trials

**DOI:** 10.2196/13253

**Published:** 2019-06-08

**Authors:** Christopher A Sanders, Stephen M Schueller, Acacia C Parks, Ryan T Howell

**Affiliations:** 1 Department of Psychological Sciences University of Missouri, Columbia Columbia, MO United States; 2 Department of Psychological Science University of California Irvine Irvine, CA United States; 3 Happify Health New York, NY United States; 4 Psychology Department San Francisco State University San Francisco, CA United States

**Keywords:** cluster analysis, depression, happiness, random allocation

## Abstract

**Background:**

A critical issue in understanding the benefits of Web-based interventions is the lack of information on the sustainability of those benefits. Sustainability in studies is often determined using group-level analyses that might obscure our understanding of who actually sustains change. Person-centric methods might provide a deeper knowledge of whether benefits are sustained and who tends to sustain those benefits.

**Objective:**

The aim of this study was to conduct a person-centric analysis of longitudinal outcomes, examining well-being in participants over the first 3 months following a Web-based happiness intervention. We predicted we would find distinct trajectories in people’s pattern of response over time. We also sought to identify what aspects of the intervention and the individual predicted an individual’s well-being trajectory.

**Methods:**

Data were gathered from 2 large studies of Web-based happiness interventions: one in which participants were randomly assigned to 1 of 14 possible 1-week activities (N=912) and another wherein participants were randomly assigned to complete 0, 2, 4, or 6 weeks of activities (N=1318). We performed a variation of *K*-means cluster analysis on trajectories of life satisfaction (LS) and affect balance (AB). After clusters were identified, we used exploratory analyses of variance and logistic regression models to analyze groups and compare predictors of group membership.

**Results:**

Cluster analysis produced similar cluster solutions for each sample. In both cases, participant trajectories in LS and AB fell into 1 of 4 distinct groups. These groups were as follows: those with high and static levels of happiness (n=118, or 42.8%, in Sample 1; n=306, or 52.8%, in Sample 2), those who experienced a lasting improvement (n=74, or 26.8% in Sample 1; n=104, or 18.0%, in Sample 2), those who experienced a temporary improvement but returned to baseline (n=37, or 13.4%, in Sample 1; n=82, or 14.2%, in Sample 2), and those with other trajectories (n=47, or 17.0%, in Sample 1; n=87, or 15.0% in Sample 2). The prevalence of depression symptoms predicted membership in 1 of the latter 3 groups. Higher usage and greater adherence predicted sustained rather than temporary benefits.

**Conclusions:**

We revealed a few common patterns of change among those completing Web-based happiness interventions. A noteworthy finding was that many individuals began quite happy and maintained those levels. We failed to identify evidence that the benefit of any particular activity or group of activities was more sustainable than any others. We did find, however, that the distressed portion of participants was more likely to achieve a lasting benefit if they continued to practice, and adhere to, their assigned Web-based happiness intervention.

## Introduction

### Background

There is a wide variety of electronic health (eHealth) and mobile health (mHealth) interventions available to anyone who is interested in Web-based mental health care or self-improvement and has access to the internet or a mobile network. The individual goals of these Web-based interventions range from smoking cessation [1] to the prevention of weight gain [2] to even the treatment of posttraumatic stress disorder and depression [3]. Many interventions, however, lack a specific focus and instead attempt to build general wellness or *happiness*. These interventions have been called elsewhere online positive psychological interventions (OPPIs) [4]. OPPIs have been the subject of much research and development, given people’s strong interest in pursuing and increasing happiness.

Although the efficacy of many of these Web-based interventions, including OPPIs, has been demonstrated in the peer-reviewed academic literature, little information is available on the sustainability of the benefits that people see when they use these interventions. Although it has been suggested that such information might be useful to allow users to make educated choices about eHealth and mHealth interventions [5], the methods to produce such an understanding have not been well developed [6]. In a review of 11 OPPI efficacy studies featuring randomized controlled trial designs, 4 studies (approximately 36%) did not report effects beyond the posttest [4]. The remaining 7 studies included in the review, however, provide initial evidence that the increases to happiness following an OPPI can be partially sustained for 6 weeks [7], 3 months [8], and 6 months [9,10] after the completion of the intervention and that a remediation of depression symptoms can similarly be observed 3 months [11] and 6 months [9,10,12,13] after exposure to an OPPI.

Although these studies do provide initial support for the idea that the benefits of an OPPI can be sustained, OPPI efficacy trials with randomized designs and some degree of follow-up assessment are limited in number beyond this subset [4] and, to the best of our knowledge, none of them have conducted specific analyses to understand the longitudinal outcomes. To address this discrepancy, in this analysis, we apply a person-centric or *idiographic* approach to post-OPPI follow-up assessments to identify which outcome trajectories are most likely before exploring individual differences in distinct longitudinal outcome trajectories.

### Online Positive Psychological Interventions and the People Who Use Them

OPPIs are most often brief, skill-based exercises that are intended to improve happiness and well-being by teaching individuals the cognitive and behavioral strategies of chronically happy people [14]. These interventions are technological translations drawing from the broader area of positive psychological interventions (PPIs) which have generally demonstrated efficacy in using a variety of strategies and delivery modalities [15,16]. Bolier and Abello [4] reviewed the evidence specifically for OPPIs and not only found that effects tended to be smaller than those found generally for offline PPIs but also noted that the only direct comparison at the time of publication between a Web-based and offline intervention found no significant difference between them [17]. Thus, it is likely that many of the benefits accrued from PPIs also apply to OPPIs.

Meta-analyses have revealed that most studies, be it Web-based or computer-based PPIs, have evaluated only the immediate impact of the intervention [15,16], comparing baseline reports of happiness with assessments of happiness made immediately following the intervention. We mentioned earlier that a smaller subset of studies have continued to collect data for 3 or more months after the completion of a given intervention [7,9-11,13,18], and these studies tend to find evidence, at the level of group-wise analyses, that many happiness interventions continue to benefit individuals who practice them through this period. This line of research, however, leaves a number of open questions regarding the nuances of longitudinal outcomes, particularly regarding individual differences.

Any person-centric approach should also appreciate the types of people who tend to seek out and use OPPIs. Fortunately, the characteristics and behaviors of such people have been investigated in past research. Parks et al [19] provide a thoughtful analysis of the type of lay people that free OPPIs appeal to, a group that the authors refer to as Web-based happiness seekers. These authors draw 3 main conclusions. First, they found that roughly half of the seekers are somewhat happy people intending to achieve greater happiness, but that the other half are quite distressed and some might even be experiencing a mental health condition. Second, they found that, overall, happiness seekers tend to frequently employ several activities in their pursuit of happiness and may persist with these activities for several months. Third, when happiness seekers are provided with easy access to a variety of (presumably) happiness-promoting activities, it is the frequency and variety of activities that they engage in that predict increases to mood and happiness.

It is also worth noting, however, that Web-based happiness seekers might be more motivated than others to increase their happiness or report their happiness as increasing. One investigation of such people found that even those exposed to hypothesized-inert psychoeducational material received boosts in happiness and well-being [20]. It is worth noting that the characteristics of Web-based happiness seekers may not be that different from those of people seeking other forms of psychological interventions on the Web and that the type of engagement with OPPIs is consistent with other eHealth and mHealth interventions in which they require a substantial degree of self-motivation and self-guidance. As such, the understanding of OPPIs and Web-based happiness seekers can help contribute to an understanding of eHealth and mHealth interventions more generally.

Other studies have explored those who self-select into PPIs, although not completely in a Web-based environment. For example, Kaczmarek et al [21] allowed college students to, voluntarily and anonymously, self-initiate a Web-based gratitude intervention after they completed a separate study. The 11.5% of participants who started the Web-based happiness intervention were more likely than their peers to express high levels of trait curiosity and endorse strong intentions to change their lifestyle. However, although Parks et al [19] found that the prevalence of depression symptoms was higher in Web-based happiness seekers than that found in the general population, Kaczmarek et al [21] found that depressive symptoms were related to a reduced tendency to start the intervention. Lyubomirsky et al [18] also conducted a similar study on positive interventions in which participants self-selected into either a study advertised as consisting of cognitive exercises or a study advertised as consisting of happiness exercises, with all participants randomly assigned to receive either a positive intervention or control exercise. In this study, they found no initial differences between the conditions on well-being, which would seem to be more in line with the findings of Parks et al [19] than those of Kaczmarek et al [21]. They did, however, find that the only people to significantly experience an increase in well-being after the intervention were those who sought out a happiness exercise in the first place and were administered a PPI (rather than a control exercise). This supports the finding of Haeck et al [20], in which it suggests that those who are motivated and interested in happiness-increasing activities might be more successful potentially because of increased effort or motivation. Bearing this in mind, it is worth examining the characteristics and behaviors of those who experience long-term benefits from PPIs, and specifically OPPIs, to better understand the mechanisms underlying the benefits.

### Hedonic Adaptation

One challenge in evaluating the long-term benefits of OPPIs is that happiness naturally fluctuates to some extent over time [22]. Furthermore, if OPPIs are to be lastingly effective, then they must overcome a psychological homeostatic process called hedonic adaptation [23,24]. Hedonic adaptation is the process through which most people revert to a previous and stable level of well-being even after significant life events and changes [25]. The earliest study noting this phenomenon was done on rare events, such as winning the lottery or having a limb amputated (ie, Brickman et al [26]), but a robust body of evidence also demonstrates this phenomenon in more common, major life events including job change (eg, was found by Chadi & Hetschko [27]), childbirth (eg, was found by Dyrdal & Lucas [28]), and marital divorce (see Kramrei et al [29] for a meta-analysis of divorce effects). In all these cases, the changes to happiness (whether positive or negative) that people saw following these events were generally temporary and usually dissipated completely within a few months or a year.

Hedonic adaptation is likely due to a combination of affective, cognitive, and motivational processes [30,31]. In the Sustainable Happiness Model, Lyubomirsky et al [32] argue that because one’s genetic set point accounts for around 50% of the variance in happiness and circumstances account for only about 10% of the variance, up to 40% of the variance in individual happiness is because of intentional activities, that is, things people think and do. These intentional activities can be teachable and are ultimately the focus of OPPIs. Sheldon et al [33] propose the Hedonic Adaptation Prevention model to suggest that hedonic adaptation is not inevitable but instead can be counteracted by intentional activity. Specifically, the Hedonic Adaptation Prevention model suggests that appreciation, surprise, variety, and intrinsic change are all mechanisms through which sustained benefits in happiness are achievable. Lyubomirsky and Layous [34] extend some of the thinking in this model by suggesting that the characteristics of both the intervention and the person contribute to the ultimate benefit received. In addition to aspects, such as variety, they also highlight issues such as dosage, social support, and triggers of the intervention as well as baseline affective states of the person as being drivers of happiness change. Their study guides this research by identifying the characteristics and behaviors that might be worth exploring in terms of mechanisms and long-term OPPI benefits.

### Primary Analyses of the Current Datasets

The data analyzed here originate from 2 large samples of Web-based happiness seekers, both of which have been reported elsewhere [19,35]. Although previous reports on these data have been restricted to the baseline characteristics of participants, immediate outcomes, and attrition, this paper reports the long-term outcomes and attempts to understand who achieves sustainable long-term benefits. The authors of the report on the first sample in our paper [19] conducted cluster analysis using baseline reports of depression symptoms, life satisfaction (LS), and affect balance (AB) and found that the participants fell into 1 of 2 groups. About half (49.5%) fell into a *distressed* group reporting low initial levels of well-being and high levels of depressive symptoms, whereas the other half (50.5%) formed a *nondistressed* cluster reporting high initial levels of well-being and low levels of depressive symptoms. Noteworthy, the *distressed* cluster had an average level of depressive symptoms of mean 26.74 (SD 10.58) on the Center for Epidemiological Studies–Depression Scale (CES-D; [36]), whereas the *nondistressed* cluster had an average level of depressive symptoms of mean 7.93 (SD 5.85) on the same scale. The authors argue that Web-based happiness seekers can be reasonably categorized into those who might be suffering from current mental health problems and those who are not and that the success of a positive intervention could be largely dependent on what category a participant belongs to.

Another report on these data was published by 2 of the authors of this paper (SMS and ACP) [35] who conducted a 6-week OPPI wherein the participants were assigned to 2, 4, or 6 weeks of intervention content or an assessment-only control condition. Participants received a new intervention each week such that the 2-week condition comprised 2 different PPIs, the 4-week condition comprised 4 different PPIs, and the 6-week condition comprised 6 different PPIs. The 2- and 4-week interventions were more effective at reducing symptoms than the control condition or the 6-week intervention by the end of the study period. Although participants in the 6-week condition did not obtain gains beyond those seen in the 4-week condition, those in the 6-week condition were more likely than other groups to continue practicing some of the exercises. In their interpretation of these mixed findings, the authors [35] explained that

“It might be that increasing the diversity of exercises leads to participants splitting their time among the techniques and not focusing on any of the techniques long enough to benefit substantially.”

Both previous papers on these data, however, were also limited by their use of nomothetic techniques to focus on the overall benefits that people obtained rather than trying to understand the trajectories of individual benefit. Although the first set of authors [19] did create clusters of *distressed* and *nondistressed* participants, this clustering was created at baseline and did not explore how information could be gained about individual differences in change trajectories.

### This Analysis

In a secondary analysis of these 2 large datasets (described by [19] and [35]), we explore longitudinal outcomes in 2 separable dimensions of happiness (LS and mood) after self-selected exposure to OPPIs. To best understand how happiness change occurs and is maintained, we adopt a person-centric approach that identifies the trajectories in happiness reports.

By examining LS and AB across the months following self-selection into a Web-based happiness intervention, we sought to address the following questions:

1. Do people typically experience adaptation after these interventions and return to baseline levels of well-being after the OPPI or are they more likely to achieve a sustainable well-being increase during this period?
2. If adaptation is prevalent yet avoidable, what can people do to prevent it?
3. How many other distinct well-being trajectories occur during this timeframe and what are their shapes?

The analysis we present here addresses these questions by identifying the most common outcome trajectories that people have reported following exposure to an OPPI, grouping people based on those trajectories and using group membership as a proxy to explore individual differences in longitudinal outcomes.

## Methods

### Recruitment

In both the samples, participants were directed to a common research portal via Web-based advertisements to participate in a research study on positive psychology exercises and a printed advertisement in Seligman’s *Authentic Happiness* (2002) book. No compensation, beyond the advertised benefit of participating in a happiness-boosting intervention, was offered to the participants. This study was approved by the Institutional Review Board (IRB) at the University of Pennsylvania under an exempt IRB as a process of continuous quality improvement, thus it was not deemed to be a clinical trial and was not registered as such.

### Participants

The 2 samples differed in the period during which data were collected. Those who enrolled between July 2006 and February 2007 appeared in Sample 1, whereas those who enrolled between February 2007 and November 2008 were represented in Sample 2.

The 2230 participants across Sample 1 (n=912) and Sample 2 (n=1318) were, demographically, very similar. Both samples contained more women than men (76.5% overall), and the 2 samples did not seem to differ in this regard (*z*=.496, *P*=.617) and were made up of people who were moderately educated (74.3% overall had a Bachelor’s degree; 70% in Sample 1 and 77.2% in Sample 2; *z*=-3.853; *P*<.001) and middle-aged (mean age 43.5 years overall; 45.3 years in Sample 1 and 42.3 years in Sample 2; *t*_2296_=5.944; *P*<.001). As described by Parks et al [19], these individuals were, in addition, likely to be distinct from other research samples because they represent the self-selected individuals who actively seek to increase their happiness through Web-based interventions and other mediums. Thus, the results of this paper were specific to this unique population: a group that includes many highly distressed persons seeking to overcome their depression symptoms.

### Procedures

On enrollment, participants provided consent and answered demographic questions along with a set of surveys relevant to their mental health and well-being. Afterward, they were randomly assigned to Web-based happiness intervention conditions that differed between samples.

#### Sample 1: Individual Interventions

Participants in Sample 1 were randomly assigned between conditions in a 14-group randomized controlled trial design. A total of 13 of these conditions represent hypothesized 1-week happiness activities (eg, writing a gratitude letter or savoring a beautiful day), whereas the final condition was based on a 1-week active control writing task used in previous studies (see [9]). Intervention instructions varied considerably by condition and are summarized in Multimedia Appendix 1.

#### Sample 2: Multiple Interventions

Participants in Sample 2 were randomly assigned between 4 conditions in which they received 0, 2, 4, or 6 weeks of positive psychology exercises, with a new exercise administered within each week of their assigned intervention. The 0 exercise condition was included as a waitlist control condition and participants could receive exercises after 6 weeks of completing assessments only. The exercises included in the experimental conditions were a subset of those to which Sample 1 was randomly assigned. Exercises were provided in a fixed order such that people in the 2-, 4-, or 6-week conditions in Sample 2 received the same content across conditions (with the variance in content being due to a variance in the intervention length). The administration order for the exercises is also provided in Multimedia Appendix 1 alongside the description of each exercise.

### Measures

Across the 2 samples, the surveys administered overlapped considerably. As such, the measures presented here were used in both the samples except where specified. Participants in both the samples completed a battery of surveys before intervention assignment, at the end of each week during the intervention period, and 1, 3, 6, and 12 months after the intervention period. These survey batteries contained the measures we described here, as well as others. Unfortunately, item-level data were not available for the calculation of scale reliability statistics in these samples. This occurred because scales were summarized by the survey software before data export and the item-level data used to create these summaries were no longer available.

#### Life Satisfaction

The Satisfaction with Life scale [37] is a global measure of LS that consists of 5 items, each with a 7 point Likert scale response (ranging from 1= *strongly disagree* to 7= *strongly agree*). Higher scores on each of the items indicate greater LS. The scale itself has demonstrated strong internal consistency, test-retest reliability, and construct validity in numerous studies (see [38] for a review). The items on this scale are relatively face-valid: “I am satisfied with my life.” and “If I could live my life over, I would change almost nothing.” are 2 examples. The metric used in this paper to assess LS is the sum of an individual’s scores on the items in this scale, with higher numbers reflecting greater satisfaction.

#### Affect Balance

The Positive and Negative Affect schedule [39] is a 20-item scale that asks participants to rate the extent to which they are currently experiencing each of the 20 different emotions on a 5 point Likert scale (ranging from 1=*very likely or not at all* to 5=*extremely*). A total of 10 items corresponded to positive emotions (eg, *enthusiastic* and *proud*), whereas the other 10 items reflected negative emotions (eg, *upset* and *nervous*). AB was calculated by summing the scores across positive items and subtracting the sum score across negative items.

#### Depression Symptoms

The CES-D scale [36] is a commonly used metric for current depression symptoms in research settings that asks participants to report the frequency with which they had experienced 20 different symptoms (eg, restlessness, loneliness, and loss of appetite) over the past week. Although the CES-D is not designed for the specific diagnosis of clinical depression, it has repeatedly demonstrated good reliability and construct validity across a wide variety of clinical [40] and nonclinical samples (see [41] for review) as a tool for identifying subclinical depression symptoms. The 20 items on this scale are each accompanied by a 4 point Likert scale response (ranging from 0=*rarely or none of the time* to 3=*most or almost all the time*) and these responses are summed to provide a proxy of depression symptom prevalence.

#### Use and Adherence

Participants in both samples were asked at each assessment after the posttest to report the number of days over the past week in which each assigned positive psychology exercise was performed (responses ranged from 0 to 7 days) and whether the specific instructions were adhered to (participants indicated *Yes* or *No* for each assigned exercise). As the number of exercises that were assigned to each participant varied within and between samples, responses to these 2 items were summed within timepoint for each participant and grouped based on the number of assigned activities before being z-transformed within each group.

### Statistical Analysis

We adopted the following 3-step analytic plan with a person-centric focus so that we might address our research questions with the greatest accuracy. We excluded all participants who completed fewer than 4 assessments of either LS or AB. The remaining missing data were deleted on a pairwise basis.

1. Trace a trajectory in well-being over time for each individual participant
2. Group similar trajectories based on their shape and identify which curve shapes are most common
3. Interpret the most common trajectory shapes and evaluate their relation to depression symptomology and use statistics

In the first step of our process, we mapped a trajectory within each person in terms of both AB and LS over the time spanning the pretest through the 3-month follow-up assessment. This provided us with our fundamental unit of analysis: a three-dimensional (3D; LS×AB×time) trajectory for each participant.

We then, in our second step, identified common trajectories in our samples through K-means cluster analysis [42] so that we might uncover the patterns of response change that naturally occur across this time span. Common trajectory shapes were identified in this way and individuals were grouped in these clusters based on their similarity with each of these shapes.

K-means cluster analysis [42] is an atheoretical approach to grouping people into K number of groups based purely on their similarity with one another. For this analysis, we performed a progressive series of K-means clustering, starting with K=1 and ending with K=10 and compared them to learn which value of K best represented our data (see the Multimedia Appendix 2 for a full description). The algorithm behind it employs prototypes to define each group, typically with the following computational steps (note also the differential usage of capitalization regarding the letter *K* to distinguish between the total number of clusters and the identification of a particular cluster).

1. Place K number of prototypes randomly within the reasonable observation window
2. Assign each subject to the prototype (k) it is closest to
3. For each k, reoptimize the prototype by defining it as the center (ie, mean) of all subjects that are assigned to it
4. Repeat steps 2 and 3 until none of the prototypes can be reasonably optimized further

**Figure 1:**
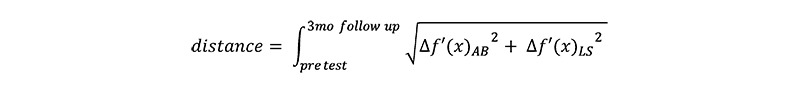
Distance function used in the clustering of participant trends. Delta symbols are used to indicate differences between prototypes and individual trends.

Once each person was described by their own polynomial trajectory in both LS and AB over time (centered at posttest; the central-most observation in both samples), we executed a series of possible K-means clustering on these trajectories using the above steps. The major deviation in our approach from the typical K-means cluster analysis was that we employed a calculus-based distance function to quantify similarities in curve shape between trajectories, rather than the squared Euclidean distance between points, when assigning participants to prototypes and when reoptimizing prototypes to better represent the participants (this distance formula represents the limited integral of a Pythagorean combination of the difference between derivative functions within each predictor dimension; see Figure 1).

This deviation represents a modification of the K-means approach to match our research objectives: We are interested in how well-being changes over time, rather than well-being itself, and the purpose of our modification is to isolate the manner with which well-being changes. *Change*, in a mathematical sense, can be found by calculating the derivative of a function; the result is a function that plots the slope of the original trajectory, in this case, across time. By clustering along the total difference between derivatives (as we have done here), we have modified the standard K-means cluster analysis to cluster well-being trajectories based only on similarities in curve shape and ignoring original function intercepts.

With each participant clustered based on curve shape, we closed our analysis with an interpretation of these newly identified longitudinal outcomes (ie, clusters) and an analysis of predictors of group membership. Observing that our clusters inadvertently seemed to differentiate based on raw self-reported levels of well-being at baseline, we employed exploratory analyses of variance (ANOVAs) to describe group-wise summaries of responses across the observation period. We then used correlation and logistic regression to explore how depression, frequency of use, and adherence to instructions might relate to the distinct longitudinal outcomes and predict group membership.

## Results

### Common Trends

We began our analytic process by fitting a within-person polynomial regression model to both LS and AB scores across the available time span. Each of these bivariate models (2 for each participant) was raised to the polynomial degree of 2 less than the number of observations to produce precise, but unsaturated, representations of change in LS or AB over time. Our combination of models resulted in a 3D (ie, LS×AB×time) trajectory representation for each individual participant (Step 1). Trajectories were then grouped based on curve shape by performing the K-means cluster analysis [42] described above that employs a customized distance function to account for functional derivative differences rather than the Euclidean distance between points (Step 2). The final clusters were then interpreted through factorial ANOVA before being classified as distinct longitudinal outcomes and compared with one another in terms of self-reported depression symptoms and use statistics (Step 3).

**Figure 2:**
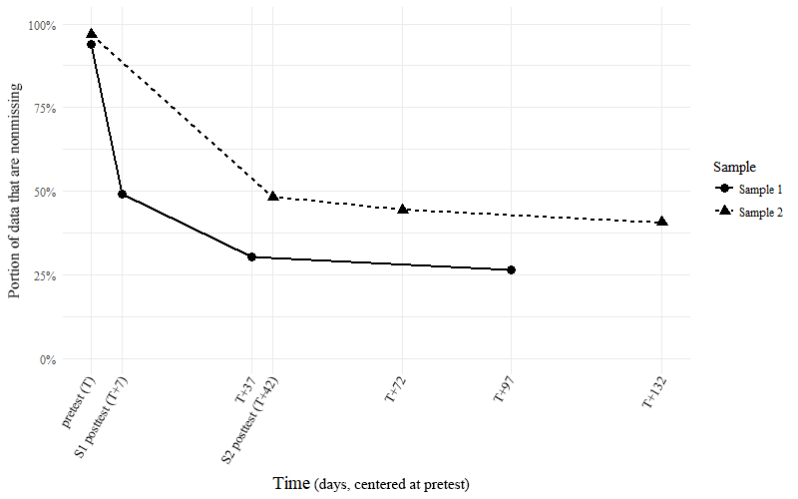
Survival curves demonstrating the portion of participants retained over time.

**Figure 3:**
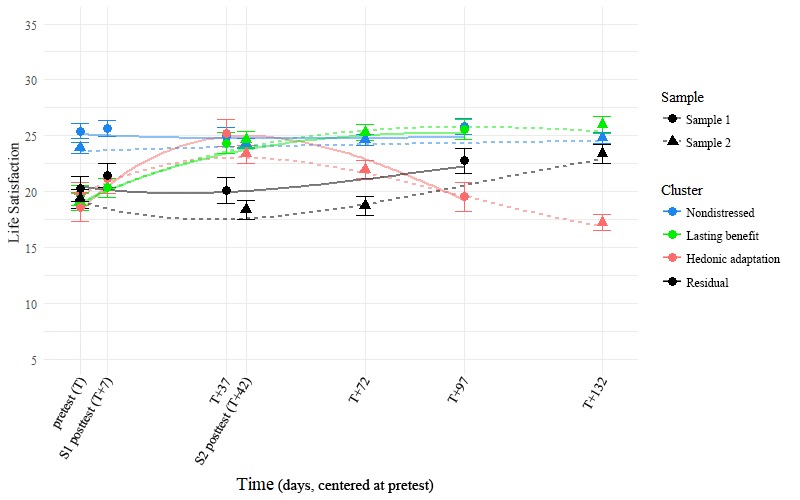
Life satisfaction trajectories over time by sample and cluster. The points in the foreground represent observed group-wise means, whereas the faded lines in the background represent the prototype trajectories that each cluster is based on. Error bars represent standard error of the mean.

The same approach was applied to both samples for confirmation and comparison purposes and all evaluations were initially conducted within the sample. Furthermore, as we discuss below and as indicated in Figures 2 and 3, similar trajectories were identified across samples in terms of both shape and position. In addition, taking into consideration the methodological and demographic similarities between the 2 samples, we decided to collapse our analysis of these variables across samples for concise presentation here except where stated.

### Attrition

The primary concern in terms of attrition or missing data was the availability of data for our first step, the within-person modeling of happiness trajectories over time. Trajectories were mapped for all participants with at least 4 (from a possible total of 6) data points in both AB and LS between the pretest and the 1 year follow-up assessment. Although this approach produced reasonable models of space between the pretest and the 3-month follow-up, it failed to reliably model the span of the following 9 months, wherein LS and AB were only assessed twice: 6 and 12 months postintervention. Despite many varied efforts, we were not able to arrive at any type of reasonable model for our data that included these time points.

These criteria reduced our sample sizes to 276 participants (30.3% of the original sample) in Sample 1 (where participants received only a 1-week intervention) and 579 participants (42.4%) in Sample 2, where the intervention was considerably more substantial. Proportionately, more participants qualified for analysis in Sample 2 than observed in Sample 1, χ^2^_3_=34.603, *P*<.001, potentially because of the difference in content between those 2 samples. Survival curves for the 2 samples are provided in Figure 2. Importantly, most of the attrition occurred between the first 2 assessments in either of the samples and this left open the possibility that the persons who dropped out did not complete, or even begin, their assigned intervention. Excluded participants reported significantly lower levels of LS (mean 20.49 [SD 8.15], n=1375), Welch’s *t*_1797_=-3.91, *P*<.001 and AB (mean 11.72 [SD 13.43], n=1382), Welch’s *t*_1913_=-5.74, *P*<.001, than the included participants (mean 21.89 and 14.92, respectively [SD 8.23 and 12.45, respectively], n=855) at pretest. They also reported a significantly higher prevalence of depression symptoms at this same time point (mean 18.22 [SD 12.97], n=1396), Welch’s *t*_1920_=5.24, *P*<.001, when compared with the participants who were retained in our analysis (mean 15.42 [SD 11.94]).

### Trajectories in Life Satisfaction and Affect Balance Across Time

**Figure 4:**
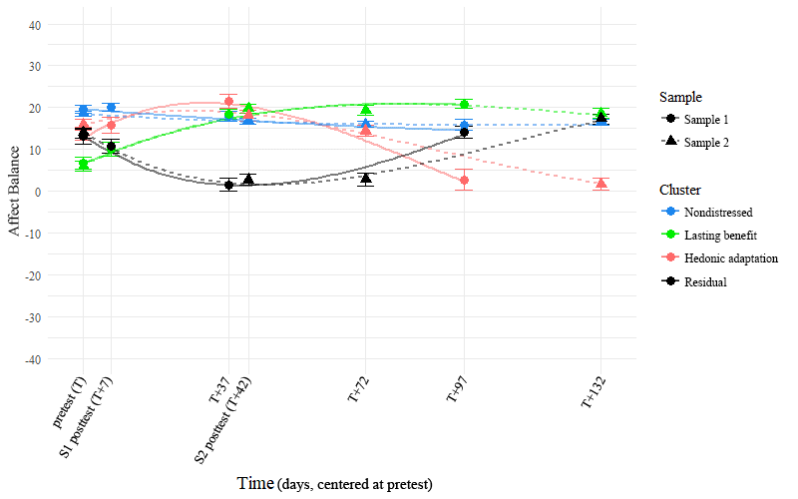
Affect balance trajectories over time by sample and cluster. The points in the foreground represent observed group-wise means, whereas the faded lines in the background represent the prototype trajectories that each cluster is based on. Error bars represent standard error of the mean.

Using Cattell’s [43] Scree approach and a descriptive comparison of the 4-factor solution to a 3- and 5-factor solution (see also Multimedia Appendix 2), we identified the 4 trajectory clusters described in this paper as being the most reasonable interpretations of trajectory shapes in the sample. These clusters are plotted in Figures 2 to 4 and were named *nondistressed* (42.8% of Sample 1 and 52.8% of Sample 2), *lasting benefit* (26.8% of Sample 1 and 18.0% of Sample 2), *hedonic adaptation* (13.4% of Sample 1 and 14.2% of Sample 2), and *residual* (17.0% of Sample 1 and 15.0% of Sample 2) based on the findings that we describe below. The reader should note that the observable similarity in common trajectory shapes across samples was not because of shared prototypes or algorithm seeds: separate and complete clustering were carried out within each of the 2 samples before displaying the results from both samples on the same plots here. Many of our subsequent comparisons were also carried out in this same way, with us occasionally reporting summary statistics across samples in the interest of brevity.

**Figure 5:**
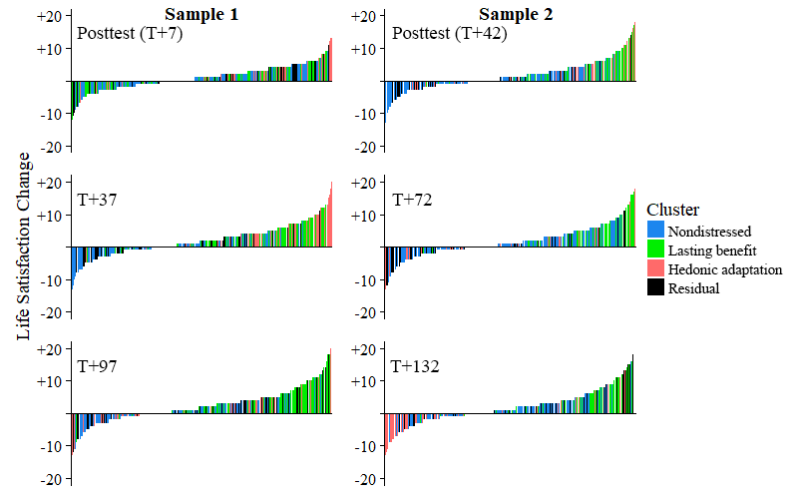
Participant-level deviation in life satisfaction from baseline by sample, timepoint, and cluster. Each bar represents one participant (arranged by value and given pairwise deletion between plots). Raw within-person differences (from pretest) are represented on the y-axis. Similar information is represented in Figure 3, though it is presented here for visual confirmation of our cluster definitions.

It is worth noting that in addition to being atheoretical, our variation on this approach ignored function intercepts (and thereby the actual position of the original data points) in our attempt to classify trajectories purely in terms of their shape. Regardless, Figures 2 and 3 show that this clustering also seems to capture distinct group-wise difference in reporting at the various time points: Once an artificial intercept is applied to each prototype, it seems to lay directly on top of the observations from the cluster that the prototype represents. Figure 5 also shows that the group-wise summary statistics displayed in Figure 3 correspond well to the distributions of responses at each time point and further confirms the precision with which we captured distinct participant trajectories.

We first examined our forecast of group-wise differences in reporting using a separate 4×4 mixed factorial ANOVA for each of the 2 outcomes, LS and AB, in each of the 2 samples. In both samples, a significant timepoint (within-participant; pretest, posttest, 1-month follow-up, and 3-month follow-up) × cluster (between-participant; nondistressed, lasting benefit, hedonic adaptation, and residual) interaction effect qualified significant main effects of both timepoint and cluster (in Sample 1: significant 2-way interactions predicted both LS, *F*_9,645_=19.67, *P*<.001, and AB, *F*_9,648_=47.31, *P*<.001; in Sample 2: significant 2-way interactions predicted both LS, *F*_9,1611_=53.412, *P*<.001, and AB, *F*_9,1611_=95.697, *P*<.001; more information is available in Supplemental Tables 1 and 2). These significant interaction effects are direct evidence of mean-level differences between groups, so we examined them further with posthoc comparisons to learn where those difference occurred.

A full posthoc analysis of all possible pairwise comparisons within samples (with Bonferroni corrections) is presented in Supplemental Tables 1 and 2. In summary, we found strong statistical evidence for exactly the patterns we would expect by observing Figures 2,3, and 4. First, a chronically high-reporting group (later identified as the nondistressed cluster; Sample 1 n=118, Sample 2 n=306) emerged across samples. As can be seen in supplemental tables 1 and 2, membership in this cluster significantly predicted higher levels of both LS and AB than the other 3 groups at the pretest assessment (T=0). Second, we observed a group of individuals in both samples who seemed to lastingly benefit from a given positive intervention (the lasting benefit cluster; Sample 1 n=74, Sample 2 n=104). Although these persons began the intervention with levels of LS and AB at or below the 3 other clusters, they showed a marked improvement in these regard when assessed a little over a month later (T+37 in Sample 1 and T+42 in Sample 2), regardless of intervention length. Furthermore, the K-means clustering approach also identified a group in each sample that showed a similar benefit around the same time point, but then displayed a hedonic adaptation curve afterward (the hedonic adaptation cluster; Sample 1 n=37 and Sample 2 n=82). Significant differences in the reporting of LS and AB between this cluster and the nondistressed and lasting benefit clusters can be observed around the 3-month follow-up in both samples. Lastly, a group of persons emerged with each clustering which we found difficult to explain in this context (the residual cluster; Sample 1 n=47 and Sample 2 n=87). Persons in this group did not seem to report an immediate benefit because of the intervention and displayed significantly less LS and AB than the other 3 groups at 3 time points: 37 (T+37; Sample 1), 42 (T+42; Sample 2), and 72 (T+72; Sample 2) days after the start of the intervention. Across separate clustering in 2 different samples, our approach arrived at what seem to be the same 4 distinct clusters of trajectories, regardless of the major differences in the interventions and timeframes between the 2 samples.

A posthoc comparison of reported LS and AB values between samples is made difficult by the fact that when the time frame is linked at the pretest (ie, when we consider the pretest assessment as our starting point), the later assessment timepoints are no longer equitable across samples. Our argument for consistency across samples in the clusters formed, however, requires some means of evaluating the similarity in cluster solutions between samples. To this end, we chose to compare reported LS and AB values between samples within each of the following 3 time point groups: early assessments (T and T+7), middle assessments (T+37 and T+42), and late assessments (T+72, T+97, and T+132; see also Supplemental Tables 1 and 2 for group-wise means and SDs by timepoint and sample). When accounting for cluster-wise and sample-wise main effects with Type 3 sums of squares, we did not observe a significant interaction effect between cluster and sample during the early assessments in terms of either LS, *F*_3, 971_=0.60, *P*=.62, or AB, *F*_3,971_=1.76, *P*=.15. This lack of a significant interaction effect was again observed within the middle assessments in terms of both LS, *F*_3,752_=0.51, *P*=.67, and AB, *F*_3,752_=1.12, *P*=.34, and again within the late assessments of LS, *F*_3,1293_=0.44, *P*=.73. A significant interaction effect between cluster and sample did emerge during the late assessments of AB, *F*_3,1293_=3.05, *P*=.028, though this effect accounted for less than 1% of the total variance in the outcome, η^2^=.006. We contend that the general lack of an observable statistical effect is not equivalent to observance of nondifference; however, we find the qualitative similarities observable in Figures 1 and 2 to be supported by this body of null findings.

**Table 1:** General binomial (logistic) models predicting membership in the lasting benefit cluster over the hedonic adaptation cluster from use statistics.

Factor		Model 1	Model 2	Model 3
Fixed effects		Intercept	Intercept	Intercept
		time^a^	time	time
		freq^b^	freq	freq
		adh^c^	adh	adh
		freq×^d^adh	freq×adh	freq×adh
		—^e^	freq×time	freq×time
		—	—	adh×time
		—	—	freq×adh×time
Model *df*		5	6	8
Log likelihood		−877.23	−873.28	−872.92
*R*-squared^f^		[.570,.641]	[.572,.644]	[.573,.645]
Akaike information criterion		1764.5	1758.6	1761.8
Residual deviance		1754.5	1746.6	1745.8
Residual *df*		1295	1294	1292
**Comparison with previous model**				
	χ^2^	—	7.9	0.73
	*df*	—	1	2
	*P* value	—	0.005	0.7

^a^Assessment time point, in days (possible values before mean centering: 7, 14, 21, 28, 35, 37, 42, 72, 97, and 132).

^b^Frequency of use (in number of days per week; scaled by total intervention length).

^c^Adherence to the specific instructions of an exercise within the past week (coded as 1=true, 0=false).

^d^Indicates an interaction effect.

^e^Not applicable.

^f^The limits of the *R*^2^ statistics presented here are Cox & Snell’s pseudo- *R*^2^ and Nagelkerke’s pseudo- *R*^2^, respectively.

**Table 2:** Optimal binomial model to describe the relationship between intervention use and the achievement of a lasting intervention benefit.

Fixed effects	Estimate (*b*)	Standard error	Standardized (β)	*z* score	*P* value
Intercept	0.47	0.11	.00	4.45	<.001
freq^a^	-0.20	0.10	-.35	-1.97	<.05
adh^b^	0.11	0.24	.09	0.45	.66
time^c^	-0.00	0.00	-.05	-0.37	.72
freq×adh	0.65	0.23	.54	2.88	.004
freq×time	0.01	0.00	.36	2.75	.006

^a^Frequency of use (scaled by intervention length).

^b^Adherence to the specific instructions of an exercise within the past week (coded as 1=true, 0=false).

^c^Assessment time point, in days (range of values before mean centering: 7, 14, 21, 28, 35, 37, 42, 72, 97, and 132).

### Relationships Between Identified Longitudinal Trajectories and Other Variables

Next, we approach the question of what might have caused some people to exhibit one pattern over another (ie, the third step of our analytic plan). In an attempt to address this question, we employed a handful of logistic regression models predicting cluster membership from metrics of depression symptomology (specifically, CES-D scores) and metrics relating to the use and continued use of the assigned activity or activities.

#### Depression

**Figure 6:**
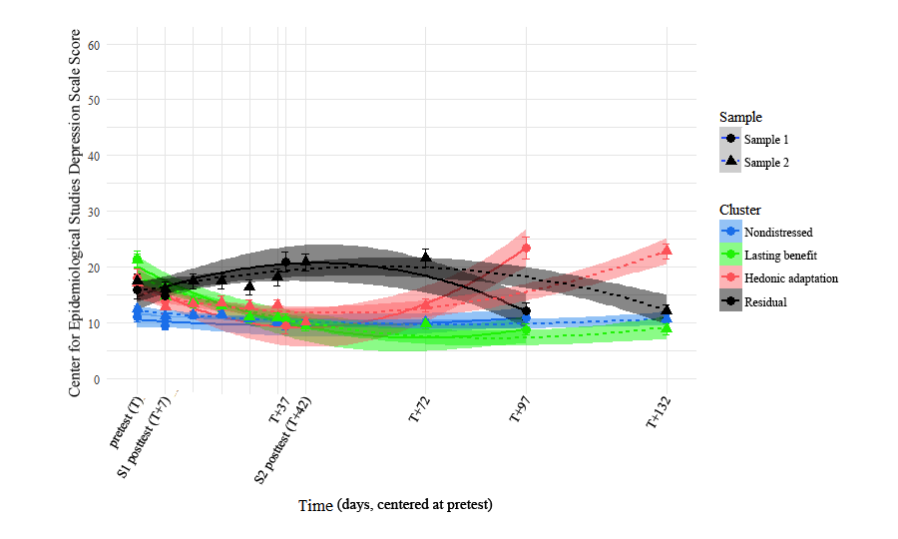
Trends in self-reported depression symptoms over time by sample and cluster. Group-wise means and standard errors are represented by points and error bars; trajectory curves are formed using a Loess smoothing function with a span width of 2 days. The standard error of the smoothing function is represented by shaded regions.

The presence of depression symptoms was assessed with the CES-D [36] at all the same timepoints where LS and AB were measured, as well as weekly during the intervention period in Sample 2. This allowed us to explore complex multidimensional multilevel models in our assessment of how this metric differentiated the 4 clusters, though the findings are relatively straightforward and can be best summarized simply by the correlations between variables: Depression was strongly negatively correlated with both LS, *r* (n=5076)=−.70, *P*<.001, 95% CI −0.71 to −0.70, and AB, *r* (n=5084)=−.75, *P*<.001, 95% CI −0.76 to −0.75, and these 2 outcomes were strongly correlated with one another, *r* (n=5076)=.62, *P*<.001, 95% CI 0.60 to 0.62. The parallels between Figures 2 and 3 are again replicated when plotting depression over this same time frame: The changes that we observe over time are not only limited to LS and AB, Figure 6 demonstrates that they also encompass large-scale changes in the presence of depression symptoms over time.

This finding emerged, in part, because of 2 factors. First, nearly half of the participants (1051/2241, 46.9% responses before deletion) began the intervention with self-reported depression symptomology that met or exceeded the common preclinical criterion for a high risk of depression (a score of 16 or above on the CES-D [36]). Second, the self-reported changes in LS and AB, similar to changes in depression symptoms, were simply more profound in distressed participants than the changes in LS and AB that occurred within seemingly nondistressed participants. With these findings, we solidified our categorization of the most popular trajectory across samples as a trajectory specific to distinctly nondistressed individuals; the remaining clusters, and the focus of our investigation are the distressed individuals among which we can more easily identify longitudinal change.

#### Frequency of Use and Adherence to Instructions

Use and adherence measures were assessed for each exercise that the participant was administered, which remained at 1 exercise for participants in Sample 1 and varied between 0 and 6 exercises in Sample 2. This became a problem for the assessment of how frequently the participant used that exercise over the past week, so these use metrics were grouped based on intervention length and z‑standardized within-group. Given our findings from the depression analysis, we employed frequency of use and adherence to instructions as possible predictors in distinguishing between participants who showed a lasting benefit because of the intervention (n=146,1206 assessments) as opposed to a hedonic adaptation curve during the assessment period (n=92,886 assessments).

In searching for an optimally fitting model to describe our data, we began with a maximal multilevel binomial model predicting a lasting benefit over hedonic adaptation and sequentially removed predictors from this model based on overall model fit. More specifically, the original maximal model predicted the binomial outcome (0=hedonic adaptation, 1=lasting benefit) from the interaction of time (days; mean centered around 28.36)×use (standardized as described above)×adherence (binomial: 0=did not adhere to the specific exercise instructions, 1=did adhere) and all lower-order interaction and main effects that comprise this 3-way interaction, with slopes and intercepts varied by participant, condition, and sample. All random effects were removed based on sequential comparisons of models, as was the 3-way interaction effect and the 2-way interaction effect between adherence and time. The final steps of this process are summarized in Table 1.

Table 1 displays summary fit statistics and comparisons between some of the latter models we considered. The outcome of each of the above functions is membership in the lasting benefit cluster (coded as *1*; functional n=1206) over membership in the hedonic adaptation cluster (coded as *0*; functional n=886). Participants were drawn from both samples and all data points observed between the pretest and the 3-months follow-up assessment are included. All predictors represented here, including time, have been grand mean centered. Model 2 is observed to be an optimal description for these data.

A closer examination of the final (optimal) model is provided in Table 2. Looking at the parameter estimates for our optimal model, we can see a number of key relationships between frequency of use, adherence to instructions, and the attainment of a lasting benefit rather than hedonic adaptation. For those who adhered to the specific intervention instructions, frequency of use considerably predicted a lasting benefit across all time points, with an especially large effect at later assessments. For those who reported that they were not adhering to the instructions of their intervention, frequency of use made relatively no impact on their possibility of membership in the lasting benefit cluster; these participants generally exhibited about a 50% probability of belonging to either group.

Table 2 presents the parameter estimates of the best-fitting model of the relationship between use statistics and membership in the lasting benefit (rather than hedonic adaptation) cluster (ie, the model labelled Model 2 in Table 1). Functional N=2092. McFadden’s *R*^2^=.387. Deviance residuals: minimum=−1.78, first quartile=−1.28, median=0.95, third quartile=1.03, maximum=1.28.

## Discussion

### Principal Findings

This analysis examined how well-being changes over time for people enrolled in an OPPI. Not surprisingly, in light of previous research showing that high baseline well-being predicts smaller OPPI effects [44], we found that the individual’s well-being at baseline had a major impact on these trajectories in our samples. Persons who were relatively well-off at the start of the intervention reported much smaller changes over time than the distinctly distressed persons who comprised roughly half of our samples. In terms of each of our specific research questions (presented in the introduction of the present paper), respectively:

1. For the distressed portion of participants, our cluster analysis of trajectories revealed that a lasting benefit following the OPPI might be just as likely as a temporary benefit (ie, adaptation); both experiences were commonly reported by participants.
2. The distressed participants who continued to use the OPPI were much more likely to see a lasting benefit rather than adaptation, especially at later time points, but only if they also reported adhering to the specific instructions of whatever OPPI they were assigned to.
3. A substantial portion of the distressed participants also exhibited trajectories that defied classification as either a lasting benefit or adaptation, though these individuals typically did not report any immediate benefit of the OPPI between the pretest and posttest. For those who were not apparently distressed at baseline, the changes to well-being over time were much subtler than the changes exhibited by distressed participants and thus more difficult to identify given the current approach.

Participants were randomly assigned between a total of 18 different conditions between the 2 samples, with 1 group representing a waitlist control condition, another group receiving a Web-based placebo intervention, and the remaining 16 experimental conditions receiving an OPPI (see Multimedia Appendix 1 for specific exercise instructions). Despite these OPPIs varying in both dosage and content, we were unable to observe any differences in participant outcomes because of condition assignment. A number of factors could explain this lack of a finding, but we contend that it is likely more because of the limitations of our design and samples rather than a general inefficacy of OPPIs. These limitations are explored further below, followed by recommendations for the design of OPPIs and OPPI studies.

### Limitations

This analysis has a number of limitations that stem from the manner with which data were collected. Our data drew from 2 samples of OPPIs, both collected from a website that appealed to those seeking to increase their happiness. Participants joined with little incentive for participation and many were recruited using an advertisement that appeared at the end of a happiness self-help book. Thus, it is hard to isolate the impact of any one specific exercise. OPPIs tend to attract motivated happiness seekers who might use additional Web-based or offline resources to boost their happiness. Our findings can also only generalize to these types of people; arguments are made here and elsewhere [19,44] as to the ways that Web-based happiness seekers differ from other members of the public. It is also worth noting, however, that this context mirrors many ways in which such interventions are deployed in real-world settings. Few incentives exist to encourage individuals to engage in such interventions; their own self-interest and happiness is just one example of something people might be interested in changing but other examples could be mental distress, physical activity, and weight.

Although our findings might suggest that users with high initial well-being derive less benefit from OPPIs, it is important to note that this pattern of findings may or may not generalize other outcomes not measured in this study. For example, high well-being users may gain resilience against future stress, or greater self-efficacy about dealing with future stress, neither of which is measured in the 2 study samples. In addition, our analysis is limited to the span of the first few months after opting into an OPPI and is based on no more than a handful of assessments per individual. Internal data collected by the third author (A. Parks) reveals that higher well-being users on an interactive well-being platform must apply more effort to achieve the same effect as their low well-being counterparts, and as a result, often take longer to reach the same level of well-being improvement. It is possible, therefore, that what appears to be a smaller effect is just an effect with longer latency or one that could be achieved if high well-being users were offered more activities to use. Finally, both samples offered participants a constrained selection of activities, which is not consistent with real-world settings, where users have many available options and can choose freely between them [45].

### Recommendations

A publication by the third author [46] outlines a number of key ways in which OPPIs and OPPI studies can be improved to maximize their efficacy and meet the ever-evolving best practices in eHealth intervention research. These findings echo support for those recommendations by demonstrating the importance of continuing to be engaged with and adherent to an OPPI long after the OPPI is learnt. Thus, we review here those recommendations that are most pertinent to user engagement and adherence.

Generally, eHealth and mHealth interventions suffer from a great deal of attrition and interventionists can potentially combat this by increasing user engagement and allowing free choice in how one self-improves [46]. The use of Web-based or mobile platforms also allows for the customization of intervention content to each user, which increases compatibility and can subsequently lead to greater intervention adherence [47]. Furthermore, the benefits of practicing an eHealth or mHealth intervention can be made apparent to the user by using technology to automatically provide quantitative feedback on a user’s progress over time and how they compare to other users. Although freedom of choice and the provision of feedback can generate problems for intervention researchers, these are the types of things that we would expect to be most beneficial to users in terms of encouraging engagement, retention, and adherence to the Web-based intervention.

There is still a great deal of work to be done in OPPI research, and we continue to echo the previous recommendations of Parks [46] as we offer guidance for this research based on these findings and their limitations. This analysis goes beyond the traditional approach of reporting average group differences to explore how participant outcomes vary across time regardless of any preconceived notion of how participants should be grouped, and we have hopefully been successful in demonstrating the value of such an approach in complementing other OPPI research. Again, attrition is a severe problem in eHealth research [48], as it is in this report, and we recommend that future investigators commit resources to aggressive email and phone follow-up with a subset of persons who drop out of a study to learn if those persons are continuing to use the intervention and utilize that information in interpreting and handling missing data. Although we acknowledge that it is important to observe dropout rates in naturalistic settings, rather than artificially driving up retention with monetary incentives, we nevertheless acknowledge the statistical difficulties that arise when large numbers of participants are missing and encourage researchers to think of creative solutions to reduce dropout and to learn more about those who do drop out.

### Conclusions

Persons suffering from moderate to severe depression symptoms are naturally attracted by advertisements for Web-based happiness-promoting intervention studies. This study provides additional evidence that OPPIs can support lasting benefits among this type of user, especially those who show regular usage. More research is needed using a greater variety of outcome measures to determine whether the impact is indeed smaller on high well-being users, or if the benefits conferred on high well-being users simply are not detectable using the currently reported outcomes. Partially explaining this recovery, those who continued to practice the activities that they received in these 2 studies continued to see gains from them 1 and 3 months after the intervention ended. The major contribution of this paper, however, is the demonstration of a novel approach within Web-based interventions to investigating long-term benefits leveraging person-centric analytic methods. Many people who might be interested in such resources would likely be interested in knowing whether such an intervention would likely benefit them. A better understanding of who benefits from Web-based interventions might also improve their use in stepped care programs where low-intensity Web-based interventions can be offered to those individuals in efforts to save cost and improve population health. Considering these findings, we advocate for the continued study of OPPIs for depressive symptoms—perhaps as an adjunct to standard treatments—and to facilitate optimal long-term outcomes, we encourage the design of OPPIs that maximize continued engagement and adherence after the intervention is over.

## Appendix

Multimedia Appendix 1 Complete listing of exercise descriptions and schedule.

Multimedia Appendix 2 Further explanation of our derivation of a four-factor solution.

## Abbreviations

AB: affect balance
ANOVA: analysis of variance
CES-D: Center for Epidemiological Studies–depression scale
eHealth: electronic health
IRB: Institutional Review Board
LS: life satisfaction
mHealth: mobile health
OPPI: online positive psychological intervention
PPI: positive psychological intervention
