# Supplementary material for: Understanding Long-Term Trajectories in Web-Based Happiness Interventions: Secondary Analysis From Two Web-Based Randomized Trials
Source: J Med Internet Res. 2019 Jun 8;21(6):e13253. doi: 10.2196/13253 (PMC6592489; doi:10.2196/13253)
Supplement: Multimedia Appendix 1 [file jmir_v21i6e13253_app1.pdf]

Appendix A

Complete Listing of Exercise Descriptions and Schedule

Exercises present in both samples (Sample 2 schedule)

- *Active-Constructive Responding* (week 1)*:* Participants learn to respond positively to good news shared by others by lengthening the conversation and helping the sharer relive the experience.
- *Gratitude Visit* (week 2)*:* Participants write a letter of gratitude and read it aloud to the target of the letter.
- *Life Summary* (week 3)*:* Participants write a summary of how they would want their life expressed to their progeny.
- *Three Good Things*(week 4)*:* Participants identify three things that went well each day and why. These good things are kept in a gratitude journal throughout the week.
- *Savoring* (week 5)*:* Participants are instructed to take time to focus intently on a positive experience 2 to 3 times each day.
- *Using Signature Strengths*(week 6)*:* Participants take the Values in Action Strengths Questionnaire and receive individualized feedback about their strengths and are instructed to use one of their top five (signature) strengths each day.

Exercises only present in Sample 1

- *ABCDE:* Participants identify and re-analyze, through writing, one pessimistic thought they've experienced each day. This writing is divided into five categories: feelings of adversity, pessimistic beliefs, consequences of these beliefs, a disputation of those beliefs, and energization that results from this dispute.
- *Shortcuts and Longcuts:* Every day for one week, they then engage in one shortcut to pleasure and one longcut to gratification, summarizing the consequences of both at the end of each day.
- *Savoring a Beautiful Day:* Participants plan a large pleasurable experience and apply a newly-learned set of explicit savoring skills to enhance their experience.
- *Finding Meaning through Strengths:* Participants learn about their five greatest strengths and employ these strengths in belonging to and to serving something larger than themselves.
- *One Door Opens, One Door Closes:* Participants write about three times in their lives when opportunities have arisen out of difficult situations. They then focus on the positive aspects of difficult situations that occur over the next week.

- *Identifying Signature Strengths:* Participants take the Values in Action Strengths Questionnaire and receive individualized feedback about their strengths.
- *You at Your Best:* Participants type up a story of a time in their lives when they were at their best, print it out, and take time each day to consider what strengths were shown during that time in their lives.
- *"Early Memories" Control Exercise:* Participants are asked to write about early memories each night.

Note. The exercise descriptions for activities present in both samples (see above) were reproduced directly from a table in S. M. Schueller and A. C. Parks' (2012) "Disseminating Self-Help: Positive Psychology Exercises in an Online Trial", *Journal of Medical Internet Research, 14*(3): e63. Participants in Sample 1 were exposed to one activity from this list for one week. Participants in Sample 2 were exposed to zero, two, four, or six weeks of content from the first section of this appendix.
