# Supplementary material for: Understanding Long-Term Trajectories in Web-Based Happiness Interventions: Secondary Analysis From Two Web-Based Randomized Trials
Source: J Med Internet Res. 2019 Jun 8;21(6):e13253. doi: 10.2196/13253 (PMC6592489; doi:10.2196/13253)
Supplement: Multimedia Appendix 2 [file jmir_v21i6e13253_app2.pdf]

Please use the following citation in reference to this document:

Sanders, C. A., Schueller, S. M., Parks, A. C., & Howell, R. T. (under review). *Understanding long-term trajectories in online happiness interventions: A secondary analysis from two web-based randomized trials* [Supplemental material]. Manuscript submitted to the *Journal of Medical Internet Research*. doi:10.2196/13253.supp

Derivation of a Four-Factor Solution

Please note that throughout this supplemental appendix, clustering was performed using a design that is ignorant of the actual values that participants reported; instead, a polynomial trend was established for each participant, first, and these trends were then clustered based solely on their similarity in terms of curve shape (i.e., the slopes of these trends over time).

Basic Description of Cluster Membership

Sample 1 Cluster *N*s, varying the *K* factor:

```
$`Cluster Sample Sizes`
$`Cluster Sample Sizes`$K3
[1] 122  78  76

$`Cluster Sample Sizes`$K4
[1] 118  74  47  37

$`Cluster Sample Sizes`$K5
[1] 107  58  47  37  27

$`Cluster Sample Sizes`$K6
[1] 81 62 47 44 36  6

$`Cluster Sample Sizes`$K7
[1] 89 41 38 37 34 27 10

$`Cluster Sample Sizes`$K8
[1] 79 44 43 33 30 21 13 13

$`Cluster Sample Sizes`$K9
[1] 82 38 30 30 27 23 22 16  8
```

Sample 2 Cluster *N*s, varying the *K* factor:

```
$`Cluster Sample Sizes`
$`Cluster Sample Sizes`$K3
[1] 363 123  93

$`Cluster Sample Sizes`$K4
[1] 306 104  87  82

$`Cluster Sample Sizes`$K5
[1] 232 151  83  66  47

$`Cluster Sample Sizes`$K6
[1] 215 118 113  59  39  35
```

```
$`Cluster Sample Sizes`$K7
[1] 141 128 105  83  57  37  28

$`Cluster Sample Sizes`$K8
[1] 168 119 118  51  37  36  30  20

$`Cluster Sample Sizes`$K9
[1] 144 132 109  48  41  40  36  20   9
```

We observe here that when the clustering is permitted to extend beyond a *K* factor of 5, some of the resulting clusters demonstrate a group membership that is low enough to lead us to seriously question the validity of those clusters as actual trends in the data, rather than a capitalization on chance variation (this is especially true in Sample 1).

Omnibus comparison of cluster solution fit, varying the *K* factor

The following two plots were incremental to the derivation of a four-factor solution in that they provided initial evidence of model fit in the four-factor solution when compared to solutions with a different number of factors. Our first analytic step in establishing the four-factor solution was an interpretation of these plots according to Cattell's (1966) Scree approach.

In each clustering, participants were assigned to the prototype that they most resembled according to the *K*-means clustering approach and the calculus-based distance function described in the primary publication. Model fit is summarized in these graphs as a total of the Euclidean variance between each participant and the prototype that they were assigned to in each clustering. Clusterings here are varied by the *K* factor, otherwise known as the number of possible atheoretical clusters permitted for each solution (e.g., a *K* of 3 tranlsates to a three-cluster solution, and so on). In the following two graphs, model fit is summarized across the LS curves and the AB curves within the same observation ranges as reported in the citing publication.

The difference in the range of the y-axis in the following two graphs is largely an artifact of a difference in the sample sizes and observation range between the two samples.

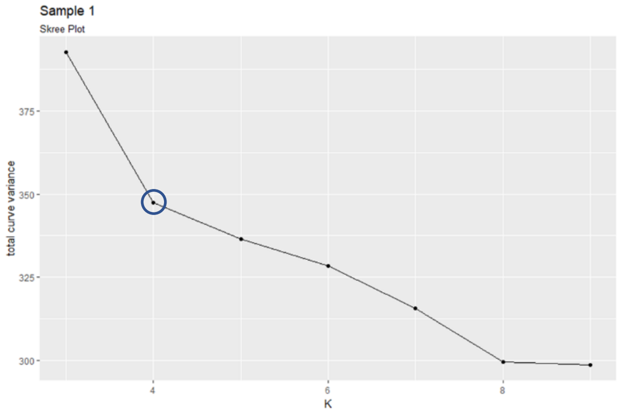

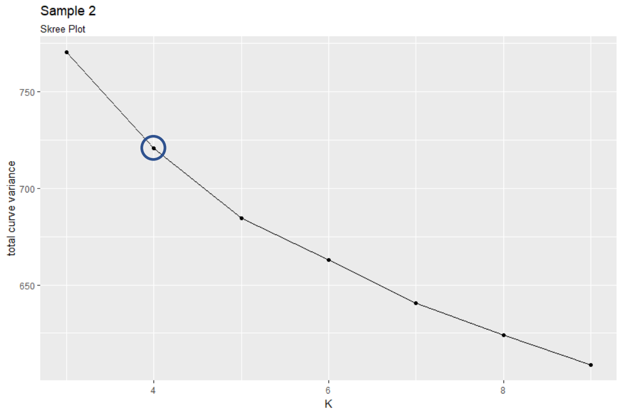

Comparison of the Three-Factor Model to the Four-Factor Model

Another approach that we employed in the comparative analysis of cluster solutions relies on comparing how individual clusters varied between cluster solutions. Specifically, we focused on how group membership differed between the four-cluster solution and the three- and five-cluster solutions. In this section, we describe our comparison of the four-factor solution to the three-factor solution. The other comparison of differential cluster membership, that between the four- and five-cluster solutions, is presented in the subsequent section.

Our primary foci in the analysis of group-membership differences between the three- and four-factor solutions are the following two contingency tables:

Sample 1

	A	B	C	*Totals*
**Non-Distressed**	82	30	6	*118*
**Lasting Benefit**	4	1	69	*74*
**Hedonic Adapt.**	36	0	1	*37*
**Residual**	0	47	0	*47*
Totals:	*122*	*78*	*76*	*276*

Sample 2

	S	T	U	*Totals*
**Non-Distressed**	300	3	3	*306*
**Lasting Benefit**	32	69	3	*104*
**Hedonic Adapt.**	31	51	0	*82*
**Residual**	0	0	87	*87*
Totals:	*363*	*123*	*93*	*579*

Given the fact that some of the cells in the above two tables hold zero values, a Chi-Square interpretation of these tables is likely to be heavily biased, so the following results should be interpreted with caution. In both samples, we observed a significant relationship between the two cluster solutions; χ^2 (6, N = 276) = 360.84, $p < .0001$, Cramer's V = 0.8085, and χ^2 (6, N = 579) = 831.84, $p < .0001$, Cramer's V = 0.8476. Specifically, in Sample 1 we observed the greatest deviations from chance between the Residual and B clusters (+254%), between the Lasting Benefit and C clusters (+239%) and between the Hedonic Adaptation and A clusters (+120%). In Sample 2, this pattern differed in that members of the Lasting Benefit and Hedonic Adaptation clusters were more likely to be grouped together in the three-factor solution; the greatest observed deviations from chance in this sample occurred between the Residual and U clusters (+523%), the Lasting Benefit and T clusters (+212%), and the Hedonic Adaptation and T clusters (+193%). In predicting membership in the three-cluster solution based on membership in our chosen solution, the four-cluster solution, we observed a significant Lambda value in both samples; λ = 0.727, 95% CI: [0.635, 0.819], in Sample 1, and λ = 0.667, 95% CI: [0.581, 0.753], in Sample 2.

Taken together, these results indicate a strong relationship, in terms of cluster membership, between the two clustering solutions. When moving from a K factor of 3 to a K factor of 4 in both samples, we primarily see two effects: an emerging distinction between the Lasting Benefit and Hedonic Adaptation clusters and an isolation of the Non-Distressed cluster.

Comparison of the Four-Factor Model to the Five-Factor Model

Our primary foci in the analysis of group-membership differences between the four- and five-factor solutions are the following two contingency tables:

Sample 1

	D	E	F	G	H	*Totals*
**Non-Distressed**	47	69	1	0	1	*118*
**Lasting Benefit**	0	28	0	0	46	*74*
**Hedonic Adapt.**	1	0	36	0	0	*37*
**Residual**	10	10	0	27	0	*47*
Totals:	*58*	*107*	*37*	*27*	*47*	*276*

Sample 2

	V	W	X	Y	Z	*Totals*
**Non-Distressed**	125	0	177	0	4	*306*
**Lasting Benefit**	0	46	53	0	5	*104*
**Hedonic Adapt.**	6	1	1	0	74	*82*
**Residual**	20	0	1	66	0	*87*
Totals:	*151*	*47*	*232*	*66*	*83*	*579*

Comparing these two cluster solutions via a chi-square analysis, and again noting the biases introduced by the empty cells in our above tables, we observed a significant dependence between the four- and five-cluster solutions; χ^2 (12, N = 276) = 548.09, $p < .0001$, Cramer's V = 0.8136 in Sample 1, and χ^2 (12, N = 579) = 1117.02, $p < .0001$, Cramer's V = 0.8019 in Sample 2. In Sample 1, the greatest deviations from chance occurred when relating the Hedonic Adaptation and F clusters (+626%), the Residual and G clusters (+487%), and the Lasting Benefit and H clusters (+265%). A similar pattern emerged in Sample 2, whereby the greatest deviations from chance were observed between the Residual and Y clusters (+566%), the Hedonic Adaptation and Z clusters (+530%), and the Lasting Benefit and W clusters (+445%). In predicting membership in the five-cluster solution based on membership in our chosen solution, the four-cluster solution, we observed a significant Lambda value in both samples; λ = 0.420, 95% CI: [0.298, 0.542], and λ = 0.398, 95% CI: [0.316, 0.480], respectively.

When moving from a cluster solution with a *K* factor of 4 to one with a *K* factor of 5, we find in both samples, that a large portion of the members in the Non-Distressed and Lasting Benefit clusters cluster together, forming the fifth cluster without causing too large of a disturbance to the other clusters. We interpret this consistent pattern of effects between samples as additional evidence of the efficacy of our clustering approach in identifying naturally-occurring trends. One possible interpretation of the new cluster that seemed to emerge from the application of a five-cluster solution (i.e., clusters E and X), in the context of the findings presented in the primary publication, is that the longitudinal effects that we reported in regards to the primarily-distressed participants might also generalize to non-distressed participants. We also observe, however, that very little information is gained by including a fifth cluster in our solution because and chose to report the four-cluster solution rather than the five-cluster solution in the interest of presenting the most coherent set of findings possible.

Conclusion

Ultimately, our decision to analyze a four-cluster solution was contingent on the size of our sample. Evidence emerged that the four-cluster solution demonstrated considerably better model fit than the three-cluster solution, especially in regard to the change in model fit between the four- and five-cluster solutions. Additionally, we learned that the inclusion of a four cluster produced a pattern of trends that was largely consistent between samples, especially in comparison to solutions with a lower *K* factor. The inclusion of a fifth cluster had a similar effect across clusters; however, our sample sizes were not large enough for a reasonable interpretation of a five-cluster solution. We chose to proceed with a four-cluster solution in the interest of maintaining statistical conservatism considering the findings presented in this report. We admit that there is a great deal of further room for exploration using a higher-order model, should future researchers choose to employ a similar analytic approach to a larger sample of OPPI-enrolled online happiness seekers.
